# On the Bite of a Rattlesnake

**Published:** 1852-08

**Authors:** S. W. Woodhouse

**Affiliations:** Academy of Natural Sciences, Philadelphia


From the Buffalo Medical Journal.

*On the Bite of a Rattlesnake.* By S. W. Woodhouse, M. D.

I received a letter from my friend Lieut. J. C. Woodruff, in which he said that you would like to receive an account of the bite of the rattlesnake, and its treatment. The only case that has come under my observation was unfortunately that of myself; this occurred whilst encamped at the Indian Pueblo of Zani, New Mexico.

The following is the extract from my journal:

Wednesday, Sept. 17, 1851. This morning Lieut. J. F. Parke, Top'l Engineers, U. S. Army, and I, were walking out to procure some specimens of birds, and when about two miles from the Pueblo, I came within a few inches of treading upon a rattlesnake, who immediately coiled himself up and got ready to strike; jumping back, I drew out my ramrod and struck him over the back with sufficient force to break it. Being a fine specimen I wished to preserve it without further injury, when placing my gun upon its head, seizing it, as I thought immediately back of the head, I picked him up, but unfortunately I had too long a hold, when he threw round his head and buried his fang in the side of the index finger of my left hand, about the middle of the first phalanx. The pain was intense, momentarily producing, as it were, a severe shock, and accompanied with much nausea. I immediately commenced sucking the wound, and at the same time got Lieut. Parke to apply a ligature round the finger to prevent the too rapid absorption of the poison. I then scarifed it freely and continued sucking until I returned to camp.

A man that was with us at the time I sent immediately back to get some aqua ammonia fort., and meet us on the road, which he did when we were about three-fourths of a mile from the town. I applied it immediately to the wound. Mr. Kern hearing what had happened, returned with him, and he wished me to try, as he said, the *Western Remedy*, that is to say, get drunk. This I had often heard of, and I was determined to try its efficacy. He was supplied with a bottle of whiskey, which I immediately commenced drinking; by the time I had arrived at the Puebla, I had drank half a pint. Already the glands in my axilla were getting sore and painful. Took some ammonia internally, scarified my finger freely and held it in a basin of warm water, which caused it to bleed freely. Then commenced drinking *brandy*, at the same time held my finger in a cup of ammonia. It took one quart of fourth-proof brandy and half a pint of whiskey (enough to have killed a man under ordinary circumstances) to produce intoxication, which only lasted four hours. During my intoxication I vomited freely; soon after my recovery from this state I removed the ligature and applied a large poultice of pulv. sem. lini. That afternoon I took ammonia internally and some pills composed of mass hydrarg. et collocynth comp., to act as a cathartic. In the evening the pain in the axilla and finger was very severe; took pulv. doveri, grs. x.

Thursday, 18th. I passed a restless night without sleep, although during the night I took at least pulv. opii, grs. iv. This morning the pain in my finger is intense, and a well-marked line of inflammation extends along the arm to the axilla. I had the entire arm and hand painted with tinct. iodine, and the flax-seed poultice renewed, commenced taking a solution of potassii iodidi as an alterative. The pills not having operated I took pulv. seidlitz, which had the desired effect. Diet, boiled rice. Several times to-day I tried to walk across the room, but each time would be seized with nausea and commenced vomiting. Took at bed-time pulv. doveri, grs. x.

Friday, 19th. I rested pretty well last night, but this morning my hand, arm, and the glands in the axilla, are much swollen and very painful.

Repeated tinct. iodine. Diet, boiled farina. Took on retiring, pulv. doveri, grs. x.

Saturday 20th. Passed a tolerable night, but my back is getting very sore, as the blankets on the stone floor make rather a hard bed. This morning the pain is very great, and the swelling down my left side as far as my hip. Renewed tinct. iodine. I am still attacked with nausea and vomiting on my attempting to walk.

I removed the skin from off my finger, and it discharged freely a watery sanguineous fluid without smell. The nail is becoming loose. The broad red line following the course of the lymphatic, is now filled with a yellowish serum. The point where the fang entered, for three-eighths of an inch in diameter, is of a dark brown color. Renewed the poultice. At bedtime took mass hydrarg. grs. v, pulv. doveri, grs. x. Continued potassii iodidi. Diet the same.

Sunday 21st. Passed a restless night, being much troubled with colic; took magnesia calc. et spts. menth pip., which relieved me, and not having my bowels open took pulv. seidlitz, which had the desired effect. Hand much swollen and filled with serum. Diet as usual.

Monday 22d. Passed a comfortable night. The swelling has left my side and arm, but little remains in the hand. I can now walk a few yards without being seized with nausea; have been sitting up most of the day. Continued potasii iodidi. Diet, mutton broth and farina.

Tuesday 23d. I awoke this morning much improved, the swelling and pain having left, with the exception of the finger, the first and second joint of which do not present a healthy appearance, the palmar surface having the appearance of gangrene, but the discharge is thin and watery, without smell. The granulations do not present a healthy appearance, they are rough, and many of them look as if they were sprinkled with yellow ochre. The nail is quite loose. Continued potassi iodidi. Diet, mutton broth, with a little of the meat.

Wednesday 24th. This day we commenced our march. I placed my hand in a sling and mounted my mule; found myself rather weak, and the mule hard to manage with but one hand; the sun was rather hot, this, with the jolting of the animal caused me to suffer considerable pain; fortunately for me, after doing six miles, we encamped. I removed the nail. From this time on the finger gradually improved. I continued renewing the poultice daily until the last of October. In the meantime there was a large slough which gradually came away and left the last phalanx exposed in two places. The granulations required occasionally the application of nitrate of silver. After this I made use of dressings of cer. simplex. Continued carrying my hand in a sling until the middle of November. A new nail commenced growing and a sinus remained open in the end of the finger; upon the introduction of the probe into the latter, the bone could be felt quite rough. A discharge from this kept up until about the 7th of February, when I removed the exfoliation of the end of the phalanx, showing evidently that the fang had entered the periosteum. Soon after this the sinus closed, leaving the finger in a deformed state, anchyloses having taken place in the first joint. The circulation is very imperfect, one of the arteries being destroyed, which renders it very susceptible of cold. The insertion of the flexor muscle has also been destroyed.

I have heard of a number of rattlesnake bites, in all of which the patient recovered if they succeeded in producing intoxication.

Dr. Fischer C. Smith, of this city, accompanied Capt. French, A. Q. M. U. S. Army, to El Paso last year, and on their return one of the teamsters was bitten by a rattlesnake, he gave him nothing but whiskey, and in three days after he was driving his team. In this case it took three pints of whiskey to produce intoxication.

Should this brief extract be of any service to you, it is at your disposal.

Academy of Natural Sciences, Philadelphia, May 22d, 1852.

